# A roadmap for the implementation of mHealth innovations for image-based diagnostic support in clinical and public-health settings: a focus on front-line health workers and health-system organizations

**DOI:** 10.1080/16549716.2017.1340254

**Published:** 2017-08-25

**Authors:** Lee Wallis, Marie Hasselberg, Catharina Barkman, Isaac Bogoch, Sean Broomhead, Guy Dumont, Johann Groenewald, Johan Lundin, Johan Norell Bergendahl, Peter Nyasulu, Maud Olofsson, Lars Weinehall, Lucie Laflamme

**Affiliations:** ^a^ Division of Emergency Medicine, Faculty of Medicine and Health Sciences, Stellenbosch University, Bellville, South Africa; ^b^ Department of Public Health Sciences, Global Health, Karolinska Institutet, Stockholm, Sweden; ^c^ Stellenbosch Institute for Advanced Study (STIAS), Wallenberg Research Centre at Stellenbosch University, Stellenbosch, South Africa; ^d^ Stockholm County Council, Forum for Health Policy, Stockholm, Sweden; ^e^ Divisions of General Internal Medicine and Infectious Diseases, Department of Medicine, University of Toronto, Toronto, Canada; ^f^ African Centre for eHealth Excellence, Cape Town, South Africa; ^g^ Health information Systems Programme, Pretoria, South Africa; ^h^ Department of Electrical and Computer Engineering, University of British Columbia, Vancouver, Canada; ^i^ British Columbia Children’s Hospital Research Institute, Vancouver, Canada; ^j^ Institute for Molecular Medicine Finland – FIMM, University of Helsinki, Helsinki, Finland; ^k^ Jumpahead AB, Stockholm, Sweden; ^l^ Department of Public Health, School of Health Sciences, Monash University, Johannesburg, South Africa; ^m^ Division of Epidemiology and Biostatistics, School of Public Health, Faculty of Health Sciences, University of the Witwatersrand, Johannesburg, South Africa; ^n^ Epidemiology and Global Health, Department of Public Health and Clinical Medicine, Umeå University, Umeå, Sweden; ^o^ Institute for Social and Health Sciences, University of South Africa, Johannesburg, South Africa

**Keywords:** mHealth for Improved Access and Equity in Health Care, mHealth, telemedicine, image-based, disgnositic support, roadmap

## Abstract

**Background**: Diagnostic support for clinicians is a domain of application of mHealth technologies with a slow uptake despite promising opportunities, such as image-based clinical support. The absence of a roadmap for the adoption and implementation of these types of applications is a further obstacle.

**Objectives**: This article provides the groundwork for a roadmap to implement image-based support for clinicians, focusing on how to overcome potential barriers affecting front-line users, the health-care organization and the technical system.

**Methods**: A consensual approach was used during a two-day roundtable meeting gathering a convenience sample of stakeholders (n = 50) from clinical, research, policymaking and business fields and from different countries. A series of sessions was held including small group discussions followed by reports to the plenary. Session moderators synthesized the reports in a number of theme-specific strategies that were presented to the participants again at the end of the meeting for them to determine their individual priority.

**Results**: There were four to seven strategies derived from the thematic sessions. Once reviewed and prioritized by the participants some received greater priorities than others. As an example, of the seven strategies related to the front-line users, three received greater priority: the need for any system to significantly add value to the users; the usability of mHealth apps; and the goodness-of-fit into the work flow. Further, three aspects cut across the themes: ease of integration of the mHealth applications; solid ICT infrastructure and support network; and interoperability.

**Conclusions**: Research and development in image-based diagnostic pave the way to making health care more accessible and more equitable. The successful implementation of those solutions will necessitate a seamless introduction into routines, adequate technical support and significant added value.

## Background

**Figure 1.**
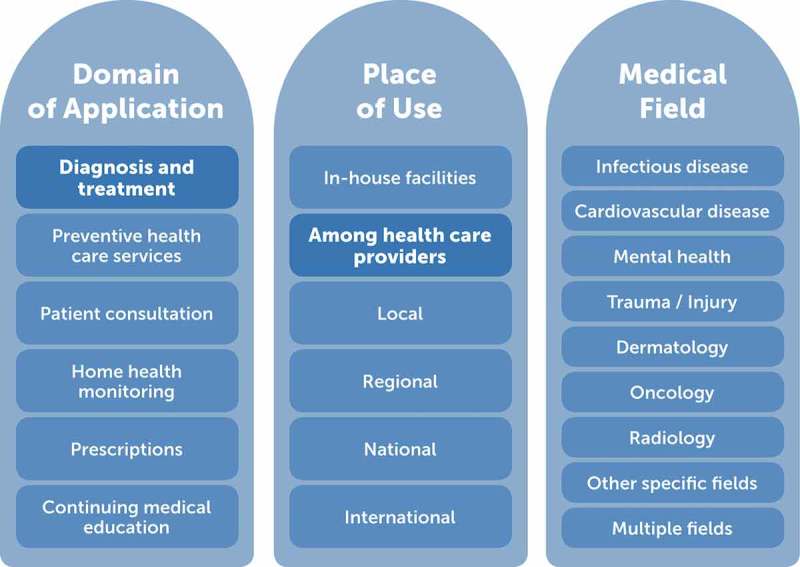
Categorization of the field of mHealth according to domain of application, place of use and medical field where mHealth is currently being applied.

mHealth technologies have transformed the world of medicine and the delivery of health-care services irreversibly and, in some instances, at a remarkably rapid pace. The field has grown so much and so quickly that it is difficult to summarize it without offering different, and often overlapping, classification alternatives. Classification can be based on the target of delivery of an app, the place where the service is aimed to be applied – in relation to the health sector as well as geographically – or the health outcome(s) that it targets (see Figure 1). The emphasis of this article is on mHealth applications for diagnostic and treatment assistance among health-care providers, regardless of the health outcome, and with a particular focus on image-based applications. This interest stems from the poor uptake of the technology [1] despite the significant gains in time, resources and quality of care that such applications could offer by remedying one of the most challenging barriers to health-care delivery in resource-scarce settings, i.e. that of access to (or outreach of) expert advice.

The most enthusiastic will claim that, in a foreseeable future, rapid developments in Information Communications Technologies (ICTs) and wireless Internet systems will jointly provide for the conception of a variety of low-cost and clinically reliable and powerful image- based apps supplying crucial health-care information, between individual clinicians or, with the developments in machine learning, in an automated fashion. In this scenario, front-line clinicians and health-care professionals will be able to receive real-time assistance for diagnosing a range of clinical conditions, and they will be provided with advice on how best to manage each situation, including treatment and referral advice. The less enthusiastic may claim that buy-in among clinicians will be poor, as it has been among patients (or laypeople) in the case of applications targeting health behaviours, with pilot projects being launched but never properly implemented and sustained. Will there be an interest for such powerful mHealth clinical tools? And will front-line practitioners trust and implement them in their routine practice? If so, under which conditions?

**Figure 2.**
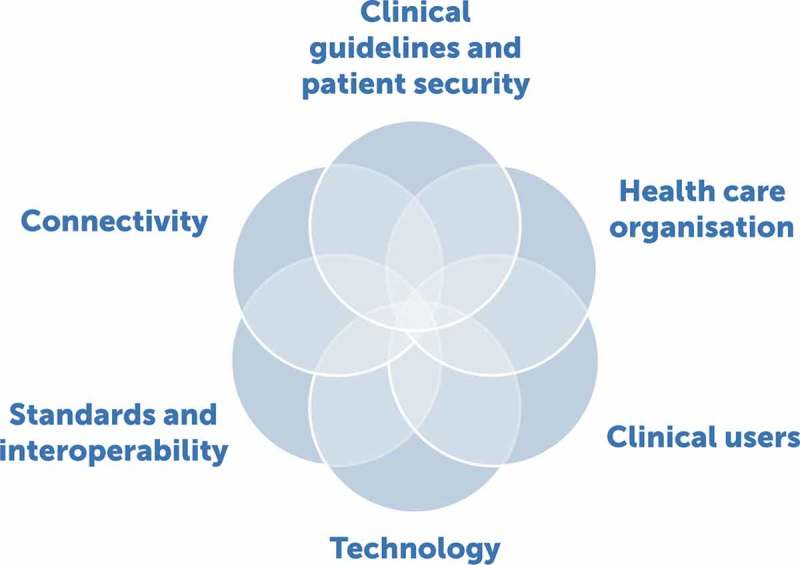
Components of a roadmap to the implementation of image-based mHealth solutions.

The method by which mHealth and other digital interventions should be implemented in clinical and health-care settings is unclear, with additional challenges when implementation is targeted for resource-scarce settings. The field needs a roadmap for innovations to be adopted and implemented as there are many foci for application, be it general [2], targeting particular segments of the population [3,4] or disease-specific [5]. While there are several clinical, technological and human aspects that such a roadmap should incorporate (e.g. Figure 2 ‘’), this article covers three of the six key components, aiming to answer the following question: What mechanisms are likely to help overcome the most important barriers to the implementation of mHealth for image-based diagnostic assistance in the clinical setting considering in turn front-line health workers (as a target group of clinical users), health-care organization and the technology (or technical environment)?

## Methods

### Procedure – a roundtable event

The investigation was embedded in a two-day roundtable meeting that gathered a mix of participants from different sectors of activities and different countries (see later in this article), in the tradition of the Stellenbosch Institute for Advanced Study (STIAS) [6]. The philosophy of the roundtables holds that consultation with interested and affected parties is an important pillar of success in many research domains. Roundtables are meant to stimulate the cross-pollination of ideas by providing a forum for discussion amongst researchers, practitioners, public- and private-sector leaders, civil-society and potential beneficiaries of research and its application or implementation.

In this roundtable, which took place in February 2017, we focused on one domain of application of mHealth, which is image-based diagnostic and treatment assistance among clinicians. The theme of the meeting was ‘Implementing image-based mobile technology for diagnostic and treatment to improve access and equity in health care’. The organizing committee consisted of a mixture of people from Sweden and South Africa, representing different fields of interest and competences. The ambition of the committee was to create the best possible conditions to draw the main line of a road map for the implementation of those mHealth solutions. The main principles followed while preparing the roundtable (a period of about one year) were:

- Draw from knowledge from studies on and applications of mHealth interventions from a diversity of domains but pay attention to their relevance for mHealth for image-based diagnosis in clinical settings
- Focus on solutions rather than problems (e.g. what to do and how to do it) for successful solutions
- Start with a general session on implementation issues and then subsequently focus remaining sessions around targeted issues for mHealth roadmaps – i.e. end users, workplace organization, technology
- Inform the discussions through short presentations from experts and specialists representing different stakeholders and broad perspectives
- Permit everyone ample opportunities to express their views through several modalities such as in interactive small group sessions, in a specific plenary and in speaker corners

### Participants

Those who participated in the roundtable were a convenience sample of people from several sectors of activity, in line with the STIAS tradition of such events. All participants took part in the event on a voluntary basis. The organizing committee was composed of stakeholders from Sweden and South Africa, who together identified a list of potential participants based on governmental agencies, public and private organizations of interest, health-app developers and individual researchers. All of them were contacted by STIAS by email, using a standardized invitation letter. On some occasions those persons contacted recommended other potential participants. In a few instances, individuals who had heard about the upcoming event asked the organizers for participation. For the STIAS events, ideally, the number of participants is expected to be somewhere between 40 to 50 with a balance among researchers, practitioners (in our case clinicians), policymakers and people from the business sector. For this roundtable, a new element was the inclusion of a number of doctoral and medical students involved in research projects on mHealth in the Western Cape (South Africa). As for previous roundtables, policymakers and business representatives were difficult to recruit and they are under-represented among the participants (see Table 1). The researchers and clinicians represented a range of disciplines and competences including medicine, nursing, engineering, public health, psychology and economics.

**Table 1.** Distribution of the participants to the roundtable by sector of activity and country.

CountrySector	RSA	Africa: Other	Sweden	Europe: Other	North and Central America
Clinical	2	5			
Clinical research	1			1	1
Research	7	1	8	4	1
Policy		4	3		
Business	4		1		1
Doctoral students			3		
Medical students			2		1

There are also usually more participants from South Africa and Sweden than from any other country or group of countries as those events are organized by representatives from Sweden and South Africa. Other African countries represented at the meeting were Uganda, Tanzania, Kenya, Malawi and Mali. From among the 55 confirmed participants, 50 actually attended the first day (36% women). It is of note that a few people heard about this upcoming event and asked to take part in it, which the organizing committee approved.

### Data-collection procedure

To determine what should be considered and what should receive priority under each overarching area, we proceeded as follows; the event itself was divided into thematic sessions of about 1.5 hours. Four of these sessions covered one aspect of the road map and highlighted the following questions.

- How should the most important barriers to the implementation of image-based mHealth in the clinical setting be overcome?
- How can front-line health-care workers be enabled to adopt image-based mHealth in their practice?
- Which are the key strategies to overcome organizational challenges to the implementation of image-based mHealth within the health sector?
- Which are the key strategies to overcome technical challenges in implementing image-based mHealth within the health sector?

**Figure 3.**
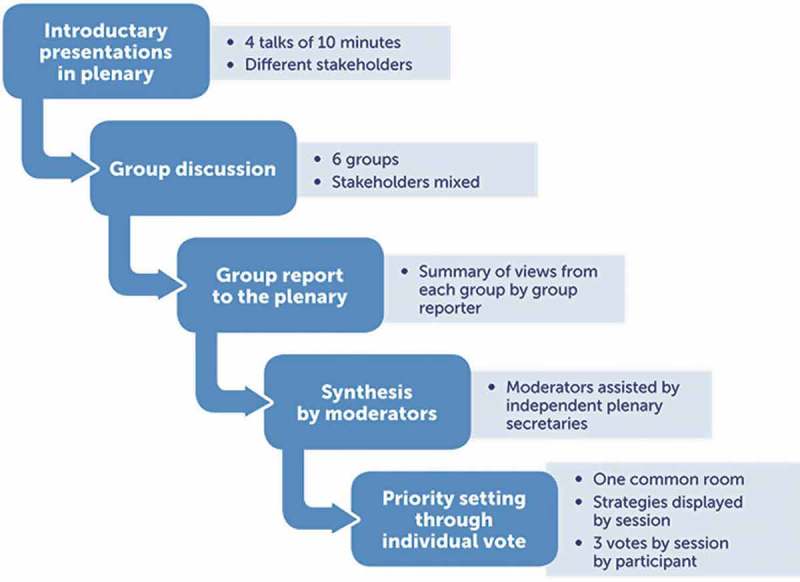
Procedure for data collection and priority setting by theme (session).

Figure 3 summarizes the steps taken to create the list of statements to be considered for priority setting. Within each session, four speakers presented one aspect relevant to the theme covered, based on the domain of expertise that he or she represented and building on facts, findings from ongoing research and reviews of findings. Directly after the presentations, the participants turned to their small-group sessions and discussed the question presented to them for 30 minutes. Each group had a chair and a rapporteur, and each discussion was assigned a note-taker on a rotational basis; all roles were assigned in advance by the organizing committee. One at a time, the rapporteurs from each table summarized what was felt to be the most relevant action items and main points. The order of the group reports changed in all sessions.

Each session was chaired by two moderators (two new ones for each session) and had two secretaries. The secretaries took notes during the rapporteurs’ presentations, and gathered the notes taken from each of the note takers during the group work. Moderators and the secretaries then met to formulate the strategies that best represented what had been expressed verbally and in writing. This involved highlighting key issues, actions and main points without losing key information. There were five to seven strategies elaborated in each session.

**Figure 4.**
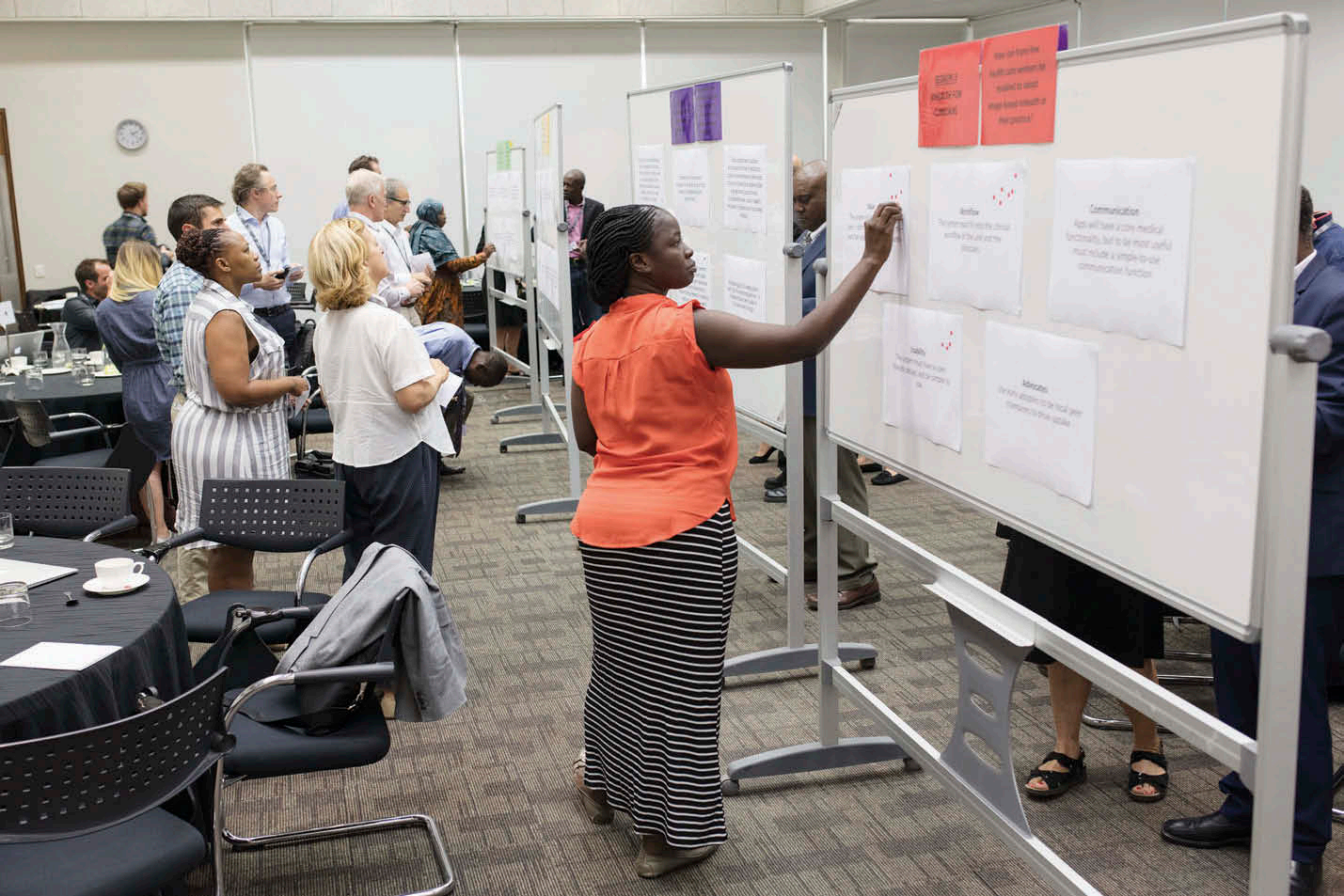
Illustration of the session where the participants proceeded to vote on the strategies they thought should receive priority, one theme at a time. The question related to each theme is at the top of the white board and the strategies proposed underneath the question. [Photo: Lisa Blom].

**Figure 5.**
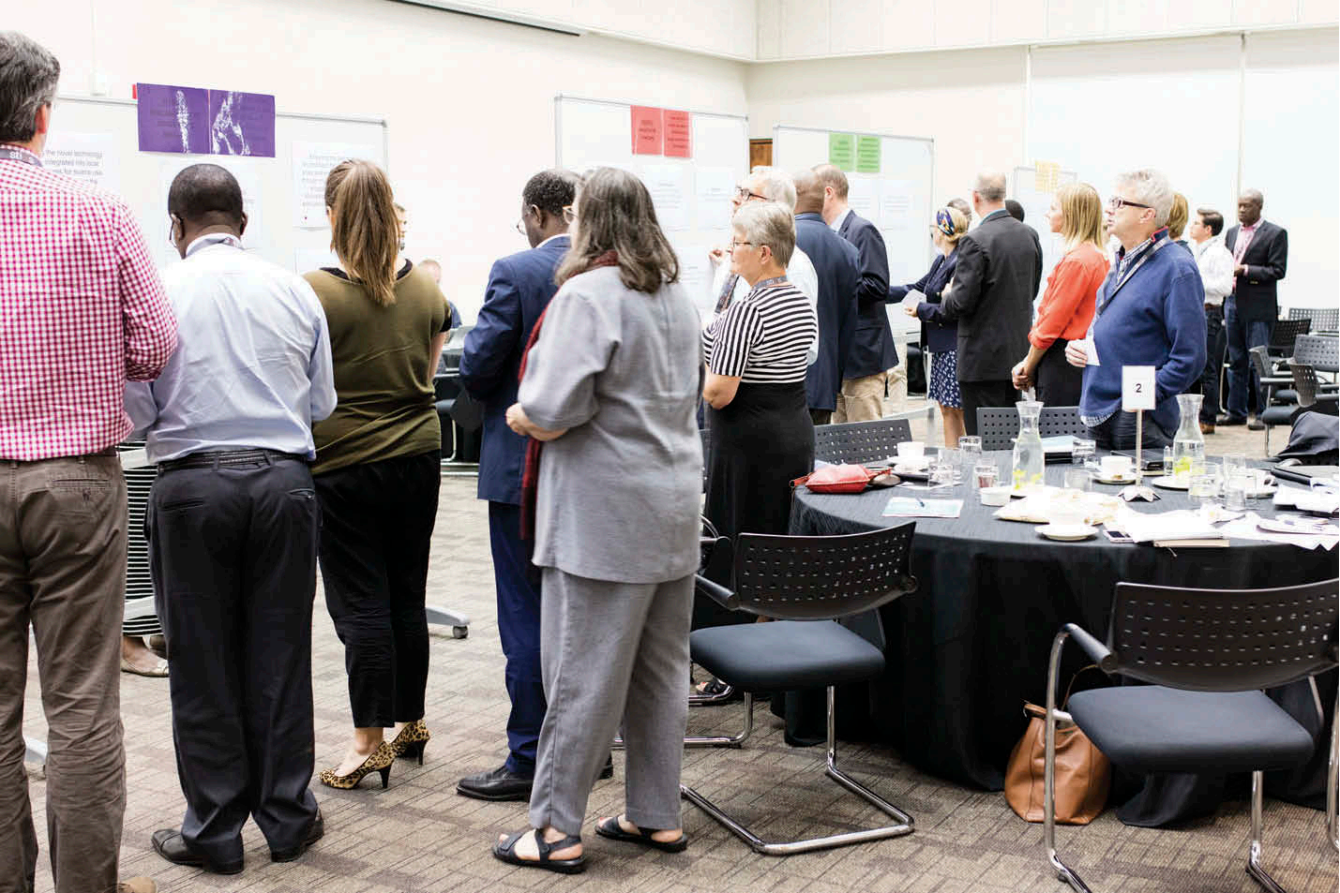
Illustration of the session where the participants proceeded to vote on the strategies they thought should receive priority, one theme at a time. The question related to each theme is at the top of the white board and the strategies proposed underneath the question. [Photo: Lisa Blom].

The strategies of each session were then printed on individual and large sheets of paper (A3 format) and placed under their respective questions on one side of a large white board (1.5 m x 1.2 m). We used four white boards – each stating one of the key questions and its related strategies. This was done in duplicate to enable participants to circulate around the room and to reduce any potential peer pressure when voting for a particular statement. When the voting session started, each participant received an envelope with 12 Post-it markers in four different colours: three markers for each session. The participants were instructed to use their markers (votes) on the strategies that they considered were most important in their own view (and context). At that moment, there were a total of 41 participants in the room. It took about 20–25 minutes for all participants to complete their votes (see Figures 4 and 5).

## Results

### Overcoming the most important barriers

Table 2 presents the six strategies derived from the session concerning how to overcome the most important barriers to the implementation of mHeath in general terms, and voting results from that session. The strategy that received the highest proportion of votes (33.6%) – i.e. the one that the participants would prioritize most – relates to the ease of the integration of the novel technology into local infrastructure. It is followed by three other strategies (each receiving roughly 20% of votes) on policy regulation/deregulation, continuous quality improvement, and the quality of the conditions for implementation.

**Table 2.** How should the most important barriers to the implementation of image-based mHealth consultation in the clinical setting be overcome? (n = 122 votes).

Strategy	Votes	
	n	%
Ensuring the novel technology can be integrated into local infrastructures for routine use (e.g. ensuring there is the appropriate electrical power, network connectivity and availability of devices).	41	33.6
Addressing policy deregulation and ‘light-touched regulations’ to implementation and scale of innovative technology.	25	20.5
Continuous quality improvement initiatives to ensure the highest practice standards are maintained over time, and mechanisms to ensure that the mHealth innovations meet (and preferably exceed) acceptable standards of care.	24	19.7
Ensuring the most suitable environment for the introduction, scale and maintenance of devices through multi-sectoral stakeholder engagement (government; clinical health care, public health, end-users; business and business models).	21	17.2
Implementation of education strategies for all users (e.g. among health care, the general public, government).	8	6.6
Ensuring that the novel technological tools meet the requisite ‘gold standard’ for implementation.	3	2.5

### Overcoming barriers among front-line health-care workers

The strategies formulated during the session concerning factors that may enable front-line health workers to overcome barriers in mHealth implementation are presented in Table 3. Although five strategies were highlighted as a result of the plenary session, three stood out – in particular the need for any system to significantly and immediately add value to the users (39.7%), usability of mHealth apps (28.9%) and goodness-of-fit into the work flow (22.3%).

**Table 3.** How can front-line health-care workers be enabled to adopt image-based mHealth in their practice? (n = 121 votes).

Strategy	Votes	
	n	%
Value – The system must add value to the users – both clinicians and patients – and that value must be immediately visible.	48	39.7
Usability – The system must have a user-friendly design and be simple to use	35	28.9
Workflow – The system must fit into the clinical workflow of the unit and the clinicians.	27	22.3
Advocates – Use early adopters to be local peer champions to drive uptake.	7	5.8
Communication – Apps will have a core medical functionality, but to be most useful must include a simple-to-use communication function.	4	3.3

### Overcoming organizational barriers in health-care services

Table 4 presents the five strategies formulated in the session concerning how to overcome organizational barriers in the health-care services. Three strategies received at least 20% of the votes with the top one being related to the need for a strategy promoting good-quality general ICT infrastructure (26.4%), closely followed by the need to involve all relevant ministries early in the process of developing or implementing mHealth tools (22.3%), and that of mHealth solutions to promote quality standards and be integrated within the local health information system (20.7%). Related to the latter strategies is the one mentioning the need of national mHealth strategies with an interoperability framework. Finally, many participants voted for the notion that organizational barriers could be lowered by the inclusion of cost-effectiveness analyses early in the development process, alongside the evaluation of diagnostic efficacy.

**Table 4.** Which are the key strategies to overcome organizational challenges to the implementation of image-based mHealth within the health sector? (n = 121 votes).

Strategy	Votes	
	n	%
mHealth initiatives should be aligned with other ICT infrastructure development strategies in a country.	32	26.4
Ensure that all relevant ministries i.e. ministries of health, technology and education are included in the process.	27	22.3
mHealth solutions should promote standards and be integrated with the local health-information system	25	20.7
A national mHealth strategy with interoperability framework is needed	20	16.5
Cost-effectiveness analyses of mHealth solutions should be included in the development process	17	14.0

### Overcoming technical barriers

Seven strategies were put forward to overcome technical challenges and these cover a wide range of aspects, from the need for basic infrastructure to be established (20.3%) to the creation of interoperability standards (5.7%) (see Table 5). The need to simultaneously understand local and regional needs, while building mHealth innovations for scale, was also viewed as an important strategy (19.5%) closely followed by a strategy that encompasses several important dimensions of image acquisition, processing and transfer (18.7%). Two additional statements deemed important included the modification of regulations to promote innovation (14.6%) and the use of multidisciplinary teams to lead development (14.6%).

## Discussion

Reports and reviews on the state of knowledge regarding mHealth in sub-Saharan Africa or in some specific countries are currently available. Some of them cover a wide span of applications (the bulk of which remain behavioural ones) [7] and others have a more specific focus, like those related to non-communicable diseases (NCDs) [8]. Studies are reviewed for their theoretical underpinning, their methodological qualities, the state of advancement of the ‘solution’ proposed and their actual results. There is a general sense that there is an increasing gap between what could be an interesting mHealth application (pilots) and what actually is implemented and sustained; the criticism as to how research helps understand this gap is severe [7,8]. Understanding how, why, for whom and in what circumstances mHealth interventions improve treatment and care is important [8].

Image-based mHealth innovations for clinicians and public-health providers have the potential to improve access and equity in health care; however, many stakeholders with complementary but distinct roles must be involved for such innovations to be successful [9]. Such stakeholders include researchers, who, for instance, will provide the evidence base necessary to determine what the end users need, how to implement innovations successfully, and predict what their expected efficacy will be. Another key stakeholder is government, who will provide infrastructures, regulatory frameworks (e.g. for the protection of health information and the definition of responsibilities), and reimbursement models to create a suitable environment to implement mHealth innovations. Health-care providers will put into place systems that help overcome individual barriers and limitations felt by (too often overloaded) front-line health workers, protect health information and clearly address responsibility issues. Finally, partnerships will also need to be created with the private sector to address business-model issues such as cost-effectiveness, marketing and supply-chain issues.

The February roundtable created a space for stakeholders from all these groups to debate key implementation issues and to propose enabling strategies. Several of the enablers identified echo those put forward in other roadmaps [2–5] and a number of them cut across the subjects that were discussed from one session to another at the roundtable.

### Theme specific strategies

The most important ‘theme-specific’ strategies resulting from the roundtable can be summarized as follows. At an overarching level *(first theme)*, it is crucial to ensure that the interface between existing infrastructures and mHealth innovations is seamless. Government policy is necessary to ensure regulations uphold data security and privacy while also creating a suitable environment to facilitate mHealth innovation and implementation. A review of studies on mHealth apps for NCDs clearly reveals that regulatory policies rank among the three most important enabling factors of their implementation alongside the availability of a stable communications network and accessible maintenance services [8]. Indeed, as health practitioners around the world begin to embrace mobile health opportunities, regulatory frameworks must develop further. This is to ensure that critical aspects such as individual privacy and confidentiality are maintained, while still supporting innovations, empowering innovators and protecting patients and end-users.

When it comes to the front-line end-users *(second theme)*, it appears clearly that disruptive modifications of the work need to be anticipated, dealt with and counterbalanced not only by acting on their acceptability and usability but also by their compatibility and added value. This is really important as a recent review shows that, in the case of NCDs, only if providers believe that mHealth interventions are useful and easy to use will mHealth ultimately contribute to improved access to care [8].

To address these issues in a structured and productive manner requires prospective appraisal of potential projects utilizing appropriate planning tools and a health-systems perspective [10]. This will help to promote project success and is particularly important for planning for scale. The WHO’s MAPS Toolkit (mHealth Assessment and Planning for Scale) [11] provides useful guidance, as does the newly released WHO guide for Planning Digital Health Mobile Interventions [12]. From another perspective, it can be of interest to investigate further the reasons for the success of consumer-oriented based apps like Viber or WhatsApp among clinicians when seeking advice [12,13] in spite of the many issues of privacy and security that they entail.

From an organizational point of view *(third theme)*, the quality of the ICT infrastructure of the health-care services in place is also seen as very important. Rather than acting in silos, the implementation of mHealth solutions should involve all relevant ministries during early stages of development and that data entered into any platform should be harmonized between the applications and the local health-information system. This will likely facilitate greater use of mHealth innovations by end-users as data entry is not duplicated. This was evaluated in earlier studies [12–14] where there are the needs of an interoperability framework and of standards for both exchange of data between systems and for storage of health-related data in local and national repositories. Cost-effectiveness analyses early in the development process, alongside the evaluation of diagnostic efficacy, were also seen as potential enabling factors. As regards the technology *(fourth theme)*, the stakeholders present at the roundtable proposed several related strategies that could help overcome technical barriers; they varied much in scope and nature with the need for basic infrastructure, the need to pay attention to the local context, and the need to be aware of the many dimensions to be considered when transferring images ranking high.

It is of note that the technical environment surrounding image-based mHealth applications actually includes many components: electricity (in grid or out of grid); mobile (high-speed) data coverage; smartphones and applications; central or cloud-based information-storage and sharing systems; scanners and picture-presentation systems; systems to support expert access to data and images stored in the cloud; and patient journal systems for diagnosis feedback and storage. Whereas some components only need to be in place for specific sites, trials or pilots, all are needed to scale a given application, and even to stimulate technical innovation and investments in mHealth technology. Also, the first four components of the technical environment for mobile health are not specific to the health sector. They are needed across a wide range of social and industrial sectors and highlight the importance of integrated planning and sharing of technology assets for optimal socio-economic return.

What basic infrastructures are available and their quality vary enormously globally and on the African continent as well as in other resource-scarce settings, both between and within countries (see some examples in Table 6). Their availability and affordability depend most often on governmental investment or regulation. The need for a technical foundation is well acknowledged in a number of African countries’ National eHealth Strategies [15–18]*** and eHealth strategies [19]. The importance of this foundation is not trivial, since they are fundamental to working in the mobile digital environment.

**Table 5.** Which are the key strategies to overcome technical challenges in implementing image-based mHealth within the health sector? (n = 123 votes).

Strategy	Votes	
	n	%
Basic infrastructure must be in place, including electricity and connectivity for mHealth to succeed	25	20.3
Understand local context but build for scale	24	19.5
By whom, how and where images are interpreted should be considered from a clinical, legal and technical point of view.	23	18.7
Light-touch regulation and policy should remove hindrances and enable innovation	18	14.6
Development should be led by a multidisciplinary team from the public and private sector, including technology experts	18	14.6
Funding should not rely on end-user to pay	8	6.5
Standards for interoperability should be set and easily available	7	5.7

**Table 6.** Examples of differences in the distribution of technical resources in the sub-Saharan Africa (SSA) region (in % of population).

Country	Malawi %	Ethiopia %	South Africa %
Electricity – urban	32	85	90
Electricity – rural	4	10	77
Internet users	9	12	52

Source: Factbook.

### Strategies across themes

Not surprisingly, the *ease of integration of the mHealth applications* into existing infrastructure was put forward not only when discussing overarching issues, but also barriers more specific to front-line users and others related to the health-care organization. Beyond acceptability and usability, some put forward not only seamlessness but also added value, in particular as regards front-line users. This is in line with the necessity expressed by a number of participants to meticulously conceive the solutions/applications envisaged and come up with solid proof of cost-effectiveness [7]. There is indeed a paucity of cost-utility and cost-effectiveness studies available within the field of mHealth [19].

An additional aspect that cuts across several themes is the *need for a solid ICT infrastructure and of a support network* to be in place. Whereas this may be challenging in specific geographic areas or health-care units, the participants underlined the necessity to look at the issue in a broader perspective and involve a wider range of stakeholders (within and outside the health sector). And there are good reasons to believe that motivation to improve the ICT infrastructure in a country – such as mobile networks – might be strengthened by alignment of mHealth initiatives with other ones in the society that would benefit from improved connectivity [15]. Local small businesses, agriculture, schools and universities are likely to benefit equally to a local, rural hospital or doctor’s office if connectivity is improved. In fact, the point has been made that ICTs can facilitate the implementation of the Sustainable Development Goals (SDGs) [20].

*Interoperability* is a third cross-cutting element worth mentioning. It applies not only to local or regional environments but also to national and cross-border ones. Interoperability is crucial for the possibility of knowledge sharing and data transfer between both organizations and countries, as well as for scaling the deployment. South Africa, for example, adds Interoperability to its list of eHealth foundation elements, and has developed a Normative Health Standards Framework [21] to help guide the sector. Another aspect of inter-operability is the potential to collect large repositories of cases with known outcome, together with images and associated clinical data. These digital repositories should preferably be accessible across institutions and nations for development of computer-assisted decision-support solutions and artificial intelligence-based algorithms.

### Strengths and limitations

Embarked upon to establish key elements of a first roadmap for the implementation of image-based mHealth applications for advice and support among clinicians, this study has a number of strengths that are worth mentioning. One is the long and knowledge-driven preparation of the event where the discussions took place, a preparation that involved a core group of stakeholders from different contexts and professions. An additional strength is the focus on those aspects that are most critical at the time of implementation of mHealth solutions, i.e. user, organization, technology and the will to discuss how barriers can be overcome and what enabling factors can be put into place. The event also managed to gather participants with diverse backgrounds – country, professional background, areas in society and so on and provided them with ample opportunities to listen, exchange and express their views and concerns based on their perspectives and contexts.

A drawback of approaches of the kind we have chosen however is that people come on a voluntary basis and represent their own perspective rather than that of their organization and the representation obtained may not be as expected. At this roundtable, as in previous ones, we had an under-representation of the business sector and of policymakers. This may have influenced the strategies put forward, if not in their definition in the priority they have received. There are several reasons that contribute to explain this under-representation. One is the predominance of researchers in the group of people that organized the meeting, which may have limited our ability to reach out to policymakers and people from the business sector from the beginning. Another reason, linked to the first one, is that by tradition, the interface between research, policy and business in meetings with themes such as the one our meeting conveyed does not unfold naturally. Policymakers and people from the business sector are not familiar with such events, may experience them as unnecessarily challenging and time consuming, and hence do not give them high priority. Eventually, approaching business organizations rather than individual companies could have been more fruitful, as the former are more used to such events. Likewise, inviting civil servants rather than ministers themselves could have provided the input that is necessary to move forward without exposing politicians to discuss sensitive questions. A third explanation relates to the very topic of the meeting, which may have lacked clarity regarding what would actually be discussed, as the subject of health-care innovation in general tends to be unclear for health policymakers and practitioners and [22]. When the topic itself will become clearer to those stakeholders and they can see their role, it may become easier to raise their interest. Inevitably, in a foreseeable future policymakers will need to take into consideration the fact that mHealth will be more or less omnipresent in health care and this will force them to engage and be proactive not least as regards mobile health education, delivery and integration [23]. And there are reasons to be optimistic in that respect as, just in the sub-Saharan Africa context, there is evidence of strong collaboration among stakeholders (2014) when implementing mHealth innovations [1].

The final wording of the strategies derived from each session was determined by the moderators of the session, assisted by plenum secretaries. The moderators had experience of the domain considered and they were instructed to remain as close as possible to what was expressed. We assumed that the strategies formulated reflected well what had been exposed at the plenary sessions, but we do not know how differently they would have been formulated should other moderators have been in charge.

An additional limitation is that we did not cover all six aspects that are relevant to the elaboration of a roadmap (Figure 2). Nonetheless, with the exception of the clinical guidelines and medical ethics, most of them were raised in the context of the chosen themes (e.g. patient security and safety, standards and interoperability, connectivity). We did not pay attention to clinical specialists themselves as a group of users, something that other roadmaps have done [5] or, for that matter, to patients.

It is not easy to assess how more concrete in the strategies proposed we could have been, given the size and the diversity of the group and also the time available, but this was not the primary aim of the process or this roadmap.

## Conclusions

Image-based diagnosis is a field of research in full expansion and the foreseeable technological developments offer great promises in making health care more accessible and thereby reducing the health divide. This applies to applications for diagnostic and treatment assistance in acute phases of disease or trauma and even, perhaps increasingly, to applications that could even provide for accurate prognosis relative to patient outcome and support decision-making on treatment. Further, the formation of joint and broadly accessible image libraries with expert annotations received strong support from the participants who saw that as a proactive strategy that could greatly contribute to both academia and industry in the era of digital medicine and artificial and augmented intelligence.

At the moment, however, the flow of financial support into the field is, to say the least, shy, and at the same time there are very few convincing proofs of concept. An additional preoccupation is that policy traditionally does not develop rapidly and, in this case, may hinder the implementation of highly cost-saving ground-breaking digital tools in the medical field that can create, among others, opportunities for reimbursement – of development costs or of digital services provided.

We may also be at a time where applications are implemented in an isolated manner, something that consumes time and efforts from all parties involved. Eventually, windows of opportunities will arise for payer–provider win–win in health systems, for instance when new applications will easily be introduced within mHealth platforms already in place: systems that are known, customized and trusted.

## Paper context

Image-based diagnostic assistance can significantly reduce the burden of disease and its unequal social distribution and impact. The absence of a roadmap for the implementation of such applications in clinical settings is an obstacle. Stakeholders gathered at a two-day roundtable helped develop such a roadmap, focusing on issues relative to front-line users and organizational and technical environments. Successful implementation of mHealth solutions necessitates a seamless introduction into routines, adequate technical support and significant added value.
